# Brain mechanisms associated with internally directed attention and self-generated thought

**DOI:** 10.1038/srep22959

**Published:** 2016-03-10

**Authors:** Mathias Benedek, Emanuel Jauk, Roger E. Beaty, Andreas Fink, Karl Koschutnig, Aljoscha C. Neubauer

**Affiliations:** 1 Department of Psychology, University of Graz, Austria, BioTechMed-Graz; 2 Department of Psychology, University of North Carolina at Greensboro, USA.

## Abstract

Internal cognition like imagination and prospection require sustained internally directed attention and involve self-generated thought. This fMRI study aimed to disentangle the brain mechanisms associated with attention-specific and task-specific processes during internally directed cognition. The direction of attention was manipulated by either keeping a relevant stimulus visible throughout the task, or by masking it, so that the task had to be performed “in the mind’s eye”. The level of self-directed thought was additionally varied between a convergent and a divergent thinking task. Internally directed attention was associated with increased activation in the right anterior inferior parietal lobe (aIPL), bilateral lingual gyrus and the cuneus, as well as with extended deactivations of superior parietal and occipital regions representing parts of the dorsal attention network. The right aIPL further showed increased connectivity with occipital regions suggesting an active top-down mechanism for shielding ongoing internal processes from potentially distracting sensory stimulation in terms of perceptual decoupling. Activation of the default network was not related to internally directed attention per se, but rather to a higher level of self-generated thought. The findings hence shed further light on the roles of inferior and superior parietal cortex for internally directed cognition.

## Introduction

Many cognitive activities (e.g., imagination) do not require sensory information processing. During these activities our attention is directed to internal representations, thereby disregarding or even suppressing interfering perceptual information^1^,^2^. This study aimed to identify the brain mechanisms responsible for maintaining an internal focus of attention, and to dissociate them from task-specific cognitive processes during internally directed cognition.

External and internal attention represent competing mental states due to limited information processing capacity^1^. Examples of internally directed cognition include planning, mental simulation, or creative idea generation^3^. These activities involve constructive processes that build on memory or previously encoded information rather than immediate sensory input^4^,^5^. Internally directed cognition can either occur spontaneously or goal-directed. Spontaneous internal cognition occurs when we are at rest, or when attention is unintentionally drawn away from a task as during mind wandering. Goal-directed internal cognition, in contrast, is part of a goal-directed activity that essentially relies on internally directed attention^6^.

Keeping our attention focused on internal processes despite constant sensory stimulation is necessary to maintain an internal train of thought^7^. It has been proposed that an internal attention focus involves a decoupling of attention from perceptual information, which serves to insulate ongoing internal processes from external interference^2^. Maintaining an internal focus of attention hence can be seen as a function of executive control^5^,^8^,^9^. Importantly, task-specific cognitive processes during internally directed cognition can be conceived as being largely independent from the attention-specific mechanisms that maintain an internal focus of attention^7^.

Cognitive neuroscience has begun to unveil the brain networks related to externally directed and internally directed attention. Externally directed attention is consistently associated with the dorsal attention network (DAN) which mainly consists of the superior parietal and intraparietal regions^10^,^11^,^12^. Moreover, occipital regions are sometimes attributed to the DAN as well, or seen to form an independent visual network^13^. The DAN supports top-down attention to expected sensory stimuli during goal-directed tasks.

In contrast, internally directed attention has been commonly associated with the default (mode) network (DN^14^,^15^). The core regions of the DN include the medial prefrontal cortex, the posterior cingulate, the medial temporal lobes, and the posterior inferior parietal cortex. The relationship of internal attention and DN is partly based on indirect inference, since the DN shows reliable task-induced deactivations during all kinds of (external) tasks (e.g.^16^), but DN activation has also been related to active mental states such as deliberate prospection and imagination tasks^17^,^18^,^19^,^20^,^21^,^22^.

For example, one study compared brain activation between a visuospatial and an autobiographical planning task^22^. They found that the visuospatial task engaged the DAN while the autobiographical task engaged the DN, and both tasks additionally activated a frontoparietal control network (FPCN). Moreover, autobiographical planning was associated with task-related coupling of FPCN and DN, whereas visuospatial planning was associated with task-related coupling of FPCN with DAN. Another study manipulated the direction of attentional within a set of visuospatial tasks rather than using different tasks for internally and externally directed cognition^23^. Internal attention was implemented by asking participants to mentally continue tasks after relevant visual information was blanked. Externally directed attention was related to higher brain activation in the parietal cortex and DN regions, and the switching between internal and external attention was shown to implicate the dorsolateral prefrontal cortex.

While the DN has been associated with internal attention in general^14^,^15^, it is also often emphasized that the DN is particularly involved in constructive, self-generated thought based on content from memory^24^,^25^,^26^. Since most internal cognition tasks crucially depend on such generative processes^20^,^27^, it is hard to keep apart attention-specific and task-specific processes when comparing brain activation between internal and external cognition. It hence remains the question to what extent the DN is actually specific to internally directed attention, or to more undirected, self-generated thought that is prevalent in internal cognition tasks.

The present fMRI study addressed this question by manipulating both the level of internally directed attention (higher for masked vs. unmasked tasks) and the level of self-generated thought (higher for divergent vs. convergent thinking), aiming to disentangle attention-specific and task-specific effects on brain activation. We hypothesized that externally directed attention would be associated with the DAN and hence superior parietal lobe activation, whereas internally directed attention should be rather associated with the inferior parietal lobe^10^,^11^. We further hypothesized that DN activity would be associated with the level of self-generated thought^24^,^25^,^26^ rather than with the direction of attention, when these factors are manipulated independently.

## Results

### Task performance

The solution rate was compared between tasks (Div, Conv) and experimental conditions (External, Internal) by means of a repeated-measures ANOVA. Participants generally solved 82% of trials. The solution rate did not differ significantly between tasks (*F*[1,31] = 1.85, *p* = 0.18; Conv: 80%, Div: 84%), but was higher in the external condition (86%) as compared to the internal condition (78%; *F*[1,31] = 7.04, *p* = 0.02, eta_p_^2^ = 0.19). There was no significant interaction between tasks and conditions in task performance (*F*[1,31] = 2.79, *p* = 0.11).

### Task-general brain activation

We first computed the task-general brain activation effects reflecting the brain activation that is common across tasks and conditions, in order to provide a reference for subsequent analyses (see Table S1 in Supplemental materials). Convergent and divergent thinking on the 4-letter prompts in both attention conditions was associated with a frontal-parietal activation pattern, involving large left-lateralized parts of middle and inferior frontal gyrus extending to the anterior insula, and of the left anterior inferior parietal lobe (aIPL). Task-positive activation further included medial parts of bilateral superior parietal lobe (SPL) and bilateral calcarine gyrus. Moreover, the tasks generally showed reduced brain activation in DN regions including the bilateral posterior inferior parietal lobe (pIPL), the right temporoparietal junction, the middle and posterior cingulate, and medial prefrontal brain regions.

### Brain activation associated with internally vs. externally directed attention

**Figure 1:**
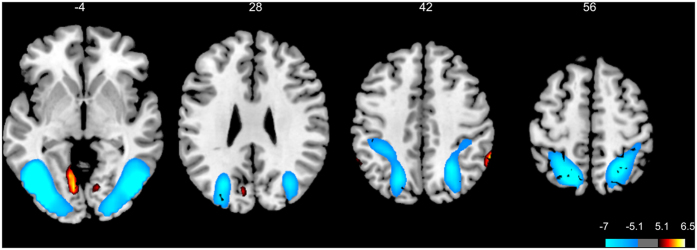
Brain activation results for internally vs. externally directed attention (Whole brain T-map, FWE-corrected at voxel-level, p < 0.05; neurological display convention). Internal attention is associated with increased activation at bilateral lingual gyrus (z = −4), left cuneus (z = 28), and right anterior IPL (z = 42), and with reduced activation at occipital and superior parietal cortex (z = −4 to 56).

The brain activation effects associated with sustained internally vs. externally directed attention are shown in Table 1 and Fig. 1. Internal attention involved increased activation in the right anterior inferior parietal lobe (aIPL) representing a posterior part of the supramarginal gyrus (SMG), in the left cuneus, and in bilateral parts of the lingual gyrus. Moreover, internally directed attention resulted in lower brain activation in extended bilateral parts of the SPL and of inferior and middle occipital regions including parts of the fusiform gyrus, areas attributed to the DAN and the visual network.

### Functional connectivity associated with internally directed attention

We explored the role of parietal regions for internally directed attention using seed-to-voxel connectivity analysis. Analyses were performed for three seeds which were centred at the parietal peak activation sites from the internal attention effect (spheres with radius of 10mm). The three seeds hence included the right aIPL which showed stronger brain activation during internally directed attention as well as left and right SPL which showed lower brain activation during internally directed attention (for peak coordinates see Table 1).

**Figure 2:**
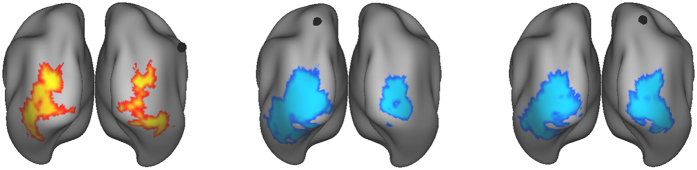
Connectivity results for internally vs. externally directed attention (Whole brain T-map, FWE-corrected at cluster-level p < 0.05). Black dots indicate position of seeds (Right anterior IPL, left SPL, and right SPL, from left to right). Warm (cold) colours indicate increased (decreased) task-related functional connectivity.

For the right aIPL seed we observed increased task-related functional connectivity with bilateral regions in the inferior and middle occipital gyrus, mainly involving visual association areas (see Table 2, and Fig. 2). In contrast, the SPL seeds both showed decreases in task-related functional connectivity with occipital brain areas, largely overlapping with those regions that showed increased connectivity with right aIPL.

### Brain activation associated with divergent vs. convergent thinking

Divergent thinking, which is argued to involve a higher degree of self-generated thought, was associated with relatively stronger activation in extended regions of the left inferior and superior frontal gyrus (IFG, SFG), the left middle temporal gyrus (MTG), the left posterior IPL (i.e., angular gyrus), the posterior cingulate, and cerebellar regions (see Table 3). There was no brain region showing stronger brain activation during convergent thinking compared to divergent thinking.

## Discussion

As expected, increased externally directed attention was associated with higher bilateral activation of occipital and SPL regions representing the visual and dorsal attention networks (DAN)^11^,^13^. The activation cluster also encompassed parts of the fusiform gyrus that is known to play an important role for word reading^28^,^29^. These findings confirm external attention recruits the DAN, and further suggests that internal attention involves reduced processing of external, visual information. More interestingly, performing a task “in the mind’s eye” involved increased activation of the right aIPL and of bilateral lingual gyrus. The aIPL region is located in the posterior part of the supramarginal gyrus (SMG). Its peak coordinates are very close (<1cm) to those previously used as parietal seed of the frontoparietal control network (FPCN)^30^, but clearly more distant ( > 2cm) from the adjacent inferior parietal parts of the ventral attention network (VAN) or the DN^13^. Internally directed attention hence did not involve increased activation of the DN, but rather seems to engage a parietal core region of the FPCN. This finding is consistent with the view that the FPCN is involved in moderating attention during goal-directed thought^22^.

In order to further elucidate the functional role of parietal regions for internally directed attention we analysed changes in functional connectivity of the significant parietal regions (i.e., aIPL and SPL) between internal and external task conditions. This analysis revealed that during internal attention conditions the right aIPL showed increased functional connectivity exclusively with inferior and middle occipital regions including regions of the secondary visual cortex and the associative visual cortex. Considering that these occipital regions showed lower brain activation during internal attention conditions, we speculate that the right aIPL may be involved in the active suppression of sensory (i.e., visual) information processing during internally directed cognition. The suppression of irrelevant external information may insulate ongoing internal processes from potentially distracting external events as proposed by the perceptual decoupling hypothesis (cf.^2^). Interestingly, internal attention conditions at the same time involved reduced connectivity between SPL and occipital regions. Taken together, internally directed attention is not only characterized by reduced activation of and coupling between DAN and visual areas, but also by increased activation of the aIPL and increased coupling with visual areas. This suggests that the right aIPL takes an active role for sustaining internally directed attention by shielding internal processes from external stimulation. The right aIPL hence may represent a key region underlying the attentional mechanism supporting perceptual decoupling^2^,^26^.

It can be argued that the internal condition involved higher memory load, since the stimulus has to be kept in mind throughout the task. This view is supported by behavioural findings showing a somewhat higher performance in the external condition. It is also in line with previous research suggesting that the inferior parietal cortex, besides many other regions, is implicated in working memory (e.g.^31^,^32^). Indeed, it can be assumed that internally directed cognition generally engages working memory, and increasing memory load may require stronger shielding of internal representations from external interference^33^. An interpretation of the aIPL in terms of working memory processes is consistent with a controlled-attention account of the aIPL and with its executive role for internally directed attention via perceptual decoupling^5^,^8^,^9^. It should be noted that differences in task difficulty can also have load-independent effects on brain activation. Task difficulty has been associated with increased recruitment of prefrontal regions and the anterior cingulate but typically not with activation of the inferior parietal cortex^34^,^35^.

Internally directed attention also resulted in increased activation of bilateral lingual gyrus and of the left cuneus. The lingual gyrus is an early visual processing area that has been associated with letter processing^36^,^37^. Moreover, both lingual gyrus and cuneus are consistently involved in visual imagery^38^,^39^. Interestingly, higher ability in divergent thinking tasks requiring visual imagination is also related to increased regional grey matter volume^40^,^41^ and lower cortical thickness^42^ specifically in the cuneus and lingual gyrus. These regions hence may be implicated in maintaining a visual representation of the stimulus after it was masked during internal attention conditions. Importantly, such a visual strategy will be more relevant during internal conditions than during external conditions, since the latter allow a reprocessing of the stimulus at any time. We assume that the lingual and cuneus activations hence reflect increased visual imagery during internal attention conditions, while the right aIPL plays a more general role for keeping attention focused on internal processes.

All tasks generally showed reduced activity of the DN when compared to a low-level baseline, which is in line with previous research on similar tasks (e.g.^43^). While DN activity was not affected by the direction of attention, we observed relatively higher DN activation when comparing divergent to convergent thinking. Divergent thinking involved increased activation of the left pIPL, the PCC, the dmPFC and the MTG, regions that are commonly attributed to the DN^13^,^44^. This finding is in line with a large number of studies that previously reported increased DN involvement in similar regions when comparing divergent and convergent thinking tasks^45^,^46^,^47^,^48^,^49^ (for reviews, see^50^,^51^). Divergent thinking tasks ask people to image novel, creative ideas for open-ended problems. While this can be generally conceived as a goal-directed task, it might be assumed to involve a considerably higher degree of constructive and undirected thought processes based on retrieval of relevant concepts from memory^52^,^53^ as compared to convergent thinking. Interestingly, core DN regions (mPFC and PCC) showed also increased activation in a decision task, when decisions were based on retrieved information rather than on perceptual input^25^,^26^. The findings hence support the view that the DN plays a unique role in the active generation of new mental representations by integrating content retrieved from memory^24^,^54^ rather than that the DN is associated with internal attention per se.

The findings of this fMRI study can be further compared with evidence from an EEG study that used essentially the same experimental design^55^. In the EEG study, increased internal attention demands induced increased EEG alpha activity particularly at posterior cortical sites. Similar EEG studies also observed significantly higher EEG alpha activity in posterior regions for tasks that are independent of external visual stimuli^56^,^57^,^58^,^59^. These posterior alpha increases thus apparently correspond to the extensive deactivations of occipital and SPL regions during internally directed attention in this fMRI study. Further relevant evidence comes from alpha connectivity studies on working memory. Internal manipulation processes were associated with functional alpha coupling between prefrontal and occipital regions, which was interpreted in terms of cognitive control exerted over visual brain regions^60^. The present fMRI findings hence show striking parallels with relevant EEG evidence. Internal attention involves increased alpha activity and reduced BOLD activation over extended posterior brain regions as well as increased functional coupling with occipital regions. Together, these findings provide converging evidence for a top-down suppression of visual processing both at the level of oscillatory and BOLD brain activity.

We conclude that the right aIPL plays an active role for the maintenance of internally directed attention by shielding ongoing internal processes from irrelevant, potentially distracting external stimulation. The DN, although commonly associated with internal cognition, however, may not be related to internal attention per se, but rather to a higher level of self-generated and potentially more undirected thought. Together, the findings shed further light on the distinct roles of inferior and superior parietal cortex (and associated brain networks including DAN, FPCN and DN) for sustained internally directed attention and self-generated thought.

## Methods

### Participants

Participants were 32 healthy, right-handed adults (19 women; mean age = 28.3, *SD* = 10.4) with normal or corrected-to-normal visual acuity and no history of mental or medical illness that could compromise cognitive functions. The study was carried out in accordance with methods that were previously approved by the ethics committee of the University of Graz. All participants gave written informed consent and were paid for participation.

### Experimental tasks and procedure

Participants worked on anagram (AN) tasks and sentence generation (SG) tasks. Stimuli to both tasks were meaningful, German four-letter words (e.g., “POST”). In the AN task, participants were required to rearrange all four letters of the stimulus to find a new, meaningful word (e.g., “STOP”). In the SG task, participants were required to generate an original, meaningful sentence by using the four stimulus letters as initial letters (e.g., “Oldies sometimes provoke tears”). In both tasks every single letter of the stimulus word had to be used exactly once, regardless of the sequence. According to common classifications^61^ the AN task represents a *convergent thinking* task, because it represents a well-defined problem and the solution space is restricted to a very limited number of (usually one) correct solutions. In contrast, the SG task is a *divergent thinking* task, because the solution space is virtually unrestricted due to the generative nature of the task (generate an original solution). Further validity evidence for the employed tasks is given by the close relationship of SG task performance with performance in other well-known divergent thinking tasks^62^, and by the association of anagram performance with cognitive intelligence^63^. We argue that the divergent thinking in the SG task involves a higher degree of self-generated thought processes than the convergent thinking in the AN task, because it requires generating new, meaningful mental representations that clearly go beyond the encoded stimulus letters. Moreover, divergent thinking might be conceived as less goal-directed and hence involve more undirected and spontaneous thinking than convergent thinking^3^.

Importantly for the aims of this study, both tasks generally rely on externally directed attention, but they can also be performed reasonably well without continuous access to the stimulus^55^. The tasks were hence performed under two experimental conditions: In the *external* condition the stimulus word remained on screen throughout the task, whereas in the *internal* condition the stimulus was masked after a brief initial encoding period. The internal condition hence enforced that the task was performed “in the mind’s eye”, thereby establishing increased internal attention demands.

In every trial, the stimulus word was presented in white capital letters on the middle of a black screen. In the *external* condition the stimulus was presented for 20 s, whereas in the *internal* condition the stimulus was shown for only 0.5 s, and then was masked by “XXXX” for the remaining 19.5 s. During this task period, participants were asked to find a solution but not to speak. In the case that participants came up with a response before the 20 s elapsed, they were instructed to keep thinking about further potential anagram solutions, or about more original sentences to ensure constant task-related activity within the task period. After this task period, the stimulus word appeared in green letters for 6 s, prompting the participants to vocalize their response. Participants were instructed to provide responses in every trial, even if they were incomplete, and to answer with “Don’t know” in case they had not solved the task in time. This enabled a close monitoring of actual performance and ensured similar response-related activity across trials. The validity of the employed experimental procedure was substantiated in a previous study^55^.

The experiment included a total of 36 task trials (18 AN tasks, and 18 SG tasks). Trials were grouped into 6 blocks à 6 trials which were organized in an ABBAAB/BAABBA fashion. Each block started with a task cue (5 s) that indicated the task type to be performed in this block (“Anagram” or “Sentence generation”). The cue was followed by 6 task trials, half of which were external and internal trials. The sequence of external and internal trials was randomly determined in each block. Trials within a run were separated by a randomly jittered fixation period (3–7 s). Additional fixation periods (10 s) were presented at the beginning and at the end of the experiment.

Before the scanner session, participants received thorough task instructions explaining the two different tasks followed by eight exercise tasks organized into two blocks à 4 tasks (2 external, 2 internal). In the scanner, a T1-scan was performed, followed by the acquisition of functional MRI data during task performance.

### fMRI data acquisition

Whole brain imaging was performed on a 3T Siemens Skyra MRI system (Siemens Medical Systems, Erlangen, Germany) using a 32-channel head coil. We acquired T1-weighted 3D-MPRAGE structural images (TR = 1560 ms, TE = 2.07 ms, flip angle = 9°, 176 sagittal slices, 1 × 1 × 1 mm, FoV = 256 × 256 mm, TI = 900 ms). BOLD-sensitive T2*-weighted functional images were acquired using a single shot gradient-echo EPI pulse sequence (TR = 2400 ms, TE = 30 ms, flip angle = 90°, 35 axial slices, 3.5 × 3.5 × 3.5 mm, distance factor 20%, FoV = 240 × 240 mm, interleaved slice ordering) and corrected online for head motion. The first two volumes were discarded to allow for T1 equilibration effects. Head motion was restricted using firm padding that surrounded the head.

Visual stimuli were presented using the software Presentation (Neurobehavioral Systems, Albany, CA), projected onto a screen, and viewed through a mirror attached to the head coil. Verbal responses were recorded by means of a MRI-compatible noise cancelling microphone (FOMRI-III; Optoacoustics, Mazor, Israel) also attached to the head coil.

### Data analysis

#### Behavioural analysis

All recorded responses were transcribed and scored. Responses were scored as correct when they represented orthographically correct solutions to the AN or SG task and all four stimulus letters were used exactly once.

#### fMRI analysis

Functional MRI data were preprocessed and analysed using SPM 12 (Wellcome Department of Imaging Neuroscience, London, UK). Preprocessing steps included slice time acquisition correction (referenced to midpoint of slice number), motion correction (interpolation with 4^th^-degree B-spline), spatial normalization into MNI space by means of the deformation field of coregistered, segmented structural data^64^, and smoothing with a 10-mm full-width at half-maximum Gaussian kernel.

Effects were estimated using the General Linear Model (GLM) as implemented in SPM 12. At the first level, four regressors of interest were included, representing the convergent thinking task (Conv; i.e. AN task) and the divergent thinking task (Div; i.e., SG task) performed either in the visible, external (External) or masked, internal (Internal) condition (i.e., Conv-External, Conv-Internal, Div-External, and Div-Internal). The four regressors were modelled with boxcar functions showing onsets 0.5 s after stimulus onsets (i.e., roughly corresponding to the onset of actual task performance after stimulus encoding, and also corresponding to the onset of stimulus masking in external conditions) and durations of 19.5 s, convolved with the canonical hemodynamic response function (HRF). Additionally, 6 motion parameters were entered as regressors of no interest. Linear contrasts were used to obtain subject-specific estimates for each effect, which were entered into a second-level analysis treating subjects as a random effect with a one-sample t-test against a contrast value of zero at each voxel.

In a first step, we analysed the task-general brain activation pattern revealing brain regions that are commonly activated during goal-directed thought across tasks and conditions (i.e., Conv-External + Conv-Internal + Div-External + Div-Internal vs. implicit baseline). In order to ensure that both tasks independently contributed to the effect, the second-level effect was doubly masked with second-level effects from single tasks (Conv-External + Conv-Internal, and Div-External + Div-Internal), hence reflecting a conjunction analysis. Establishing the task-general brain activation helps to interpret condition-specific and task-specific findings relative to general effects of task performance. The effect of *internal attention* was analysed with a contrast of internal vs. external conditions (i.e., Conv-Internal + Div-Internal vs. Conv-External + Div-External). Again, this condition effect was doubly masked with second-level effects observed for single tasks (i.e., Conv-Internal vs. Conv-External, and Div-Internal vs. Div-External) to reveal only effects that are reliable across tasks. Finally, the effect of *self-generated thought* was analysed with a contrast of divergent thinking vs. convergent thinking across conditions (Div-External + Div-Internal vs. Conv-External + Conv-Internal), doubly masked with condition-specific effects (i.e., Div-External vs. Conv-External, and Div-Internal vs. Conv-Internal).

Findings of the whole-brain analyses are reported when they are significant at voxel-level (*p* < 0.05, FWE-corrected for multiple comparisons) and cluster size is k ≥ 3. Additionally, whole-brain effects were inclusively masked with binary masks obtained from second-level effects for single tasks/conditions (*p* < 0.001, uncorrected), as well as a grey matter mask estimated based on the SPM12 gray matter tissue map (x > 0.05).

#### Functional connectivity analysis

We assessed functional connectivity changes associated with internally directed attention by means of the psycho-physiological interaction (PPI) method. This connectivity analysis was performed with the SPM toolbox *Conn* (v15)^65^. Analyses were based on the preprocessed functional data and coregistered structural data as available from the brain activation analysis. For each participant, Conn implemented CompCor, a method for identifying principal components associated with segmented white matter (WM) and cerebrospinal fluid (CSF)^66^. These components were entered as confounds along with realignment parameters in a first-level analysis. Additionally, the main effects of the psychological factors (i.e., external and internal, respectively) were entered as confounds. Task-modulation effects were computed by means of bivariate regression. We used functionally defined ROIs in inferior and superior parietal lobe defined by spheres (10 mm; masked with gray matter mask) at the peak coordinates observed in the Internal vs. External contrast analysis. At second-level, functional connectivity changes associated with the main contrast Internal vs. External were analysed and findings reported when significant at cluster-level-level (*p* < 0.05, FWE-corrected) using a cluster-forming threshold of *p* < 0.001.

## Additional Information

**How to cite this article**: Benedek, M. *et al*. Brain mechanisms associated with internally directed attention and self-generated thought. *Sci. Rep*. **6**, 22959; doi: 10.1038/srep22959 (2016).

## Supplementary Material

Supplementary Information

## Figures and Tables

**Table 1:** Brain activation for internally vs. externally directed attention.

Region	Lat.	BA	Peak (MNI)			k	*T*	*p*
			x	y	z			
Internal > External								
aIPL (SMG)	R	40	61	−46	42	8	6.73	.001
Lingual G	L	18	−10	−70	−7	40	6.68	.002
Lingual G	R	18	12	−74	−4	5	5.78	.013
Cuneus	L	18	−10	−77	28	3	5.77	.013
Internal < External								
IOG, MOG	L	19, 37	−45	−74	−7	985	11.06	<0.001
SPL	L	7	−20	−63	56	l.m.	9.36	<0.001
SPL	R	7	26	−56	53	792	11.12	<0.001
IOG, MOG	R	19, 37	47	−67	−4	l.m.	9.66	<0.001

Notes. Lat. = Laterality, BA = Brodmann area, k = cluster size, L/R = Left/right, aIPL = anterior Inferior Parietal Lobe, SMG = Supramarginal Gyrus, SPL = Superior Parietal Lobe, IOG = Inferior Occipital Gyrus, MOG = Middle Occipital Gyrus, G = Gyrus, l.m. = local maximum.

**Table 2:** Seed-to-voxel functional connectivity analysis.

Seed	Region	Lat.	BA	Peak (MNI)			k	*T*	*p*
				x	y	z			
*Right aIPL*	IOG, MOG	L	18,19	−31	−84	18	266	5.14	<0.001
	IOG, MOG	R	18,19	54	−70	−7	179	5.42	<0.001
*Left SPL*	IOG, MOG	L	18,19	−41	−91	11	617	−6.33	<0.001
	IOG, MOG	R	18,19	33	−95	14	73	−4.50	0.01
*Right SPL*	IOG, MOG	L	18,19	−20	−91	4	444	−5.94	<0.001
	IOG, MOG	R	18,19	29	−84	18	313	−5.61	<0.001

Notes. Lat. = Laterality, BA = Brodmann area, k = cluster size, L/R = Left/right; IOG = Inferior Occipital Gyrus, MOG = Middle Occipital Gyrus.

Task-related connectivity changes for the contrast Internal > External (Positive and negative T-values indicate connectivity increases and decreases, respectively).

**Table 3:** Brain activation results for divergent vs. convergent thinking (corresponding to a higher vs. lower level of self-generated thought).

Region	Lat.	BA	Peak (MNI)			k	*T*	*p*
			x	y	z			
Divergent > Convergent								
SFG	L	9	−13	49	39	16	6.22	0.004
IFG	L	47	−38	25	−7	1179	8.06	<0.001
dmPFC (MFG/SFG)	L	8,9	−6	11	63	l.m.	7.31	<0.001
MTG	L	21,22	−48	−39	−4	57	6.92	0.001
PCC	L	29	−6	−46	4	14	5.65	.01
pIPL (AG)	L	39	−52	−63	21	3	5.43	0.02
Cerebellum	L		29	−60	53	3	5.47	0.02
Cerebellum	R		36	−42	−35	188	8.43	<0.001
Cerebellum	R		15	−49	−42	8	5.52	0.02
Cerebellum	R		12	−84	−28	3	5.19	0.04
Divergent < Convergent								
–								

Notes. Lat. = Laterality, BA = Brodmann area, k = cluster size, L/R = Left/right, SFG = Superior Frontal Gyrus, IFG = Inferior Frontal Gyrus; MFG = Middle Frontal Gyrus; dmPFC = dorsomedial Prefrontal Gyrus; MTG = Middle Temporal Gyrus; PCC = Posterior Cingulate Cortex; pIPL = posterior Inferior Parietal Lobe, AG = Angular Gyrus, l.m. = local maximum.
