# Supplementary material for: Brain mechanisms associated with internally directed attention and self-generated thought
Source: Sci Rep. 2016 Mar 10;6:22959. doi: 10.1038/srep22959 (PMC4785374; doi:10.1038/srep22959)
Supplement: Supplementary Information [file srep22959-s1.doc]

SUPPLEMENTAL MATERIAL

**Brain mechanisms associated with internally directed attention and self-generated thought.**

Mathias Benedek, Emanuel Jauk, Roger E. Beaty, Andreas Fink, Karl Koschutnig,
& Aljoscha C. Neubauer

Table S1

Task-general brain activation (across tasks and conditions)

|  |  |  | **Peak (MNI)** | | |  |  |  |
| --- | --- | --- | --- | --- | --- | --- | --- | --- |
| **Region** | **Lat.** | **BA** | **x** | **y** | **z** | **k** | ***T*** | ***p*** |
| *Task-positive activation* |  |  |  |  |  |  |  |  |
| MFG | L | 6,9 | -6 | 14 | 49 | 295 | 10.25 | <.001 |
| IFG | L | 44 | -45 | 25 | 25 | 386 | 10.13 | <.001 |
| Ant. Insula | L | 13 | -31 | 21 | 0 | 40 | 9.27 | .001 |
| Ant. Insula | R | 13 | 33 | 25 | 0 | 32 | 8.65 | .001 |
| aIPL, SPL | L | 40, 7 | -45 | -39 | 46 | 419 | 10.25 | <.001 |
| SPL | R | 7 | 22 | -60 | 60 | 37 | 7.88 | .001 |
| Calcarine G | L | 17 | -13 | -91 | -4 | 6 | 5.90 | .015 |
| Calcarine G | L | 17 | -17 | -98 | 4 | 3 | 6.45 | .024 |
| Calcarine G | R | 17 | 15 | -91 | 0 | 18 | 7.47 | .004 |
|  |  |  |  |  |  |  |  |  |
| *Task-negative activation* |  |  |  |  |  |  |  |  |
| mPFC | L/R | 10 | 5 | 56 | 0 | 4 | 7.59 | .02 |
| Precentral G | R | 6 | 54 | 0 | 11 | 11 | 6.55 | .009 |
| Precuneus, PCC | L/R | 31 | -6 | -56 | 28 | 230 | 8.01 | <.001 |
| pIPL (AG) | L | 39 | -62 | -53 | 32 | 54 | 9.54 | <.001 |
| TPJ (IPL, STG) | R | 39, 40, 22 | 61 | -56 | 21 | 495 | 8.04 | <.001 |

Notes. Lat. = Laterality, BA = Brodmann area, k = cluster size, L/R = Left/right, MFG = Middle Frontal Gyrus, IFG = Inferior Frontal Gyrus; Ant. Insula = Anterior Insula; s; PCC = Posterior Cingulate Cortex; pIPL = posterior Inferior Parietal Lobe, AG = Angular Gyrus, TPJ = Temporoparietal junction; STG = Superior Parietal Lobe; l.m. = local maximum.
